# Optimizing XGBoost via mSMA_plus: A Novel Meta-Heuristic Approach for High-Precision Multiclass Dry Bean Classification

**DOI:** 10.3390/biomimetics11060379

**Published:** 2026-06-01

**Authors:** Nadir Subaşi

**Affiliations:** Department of Computer Programming, Vocational School of Technical Sciences, Kirklareli University, 39100 Kirklareli, Türkiye; nadir.subasi@klu.edu.tr

**Keywords:** dry bean classification, XGBoost, meta-heuristic algorithms, mSMA_plus, hyperparameter optimization, smart agriculture

## Abstract

Precise classification of dry bean varieties holds critical importance for agricultural sustainability, food security, and the preservation of seed quality standards. Traditional classification methods rely on human intervention and exhibit significant error rates; this necessitates the use of high-performance machine learning models and effective optimization strategies. This study aims to propose an innovative framework that optimizes the hyperparameters of the Extreme Gradient Boosting model for classifying seven different bean varieties on the Dry Bean Dataset using meta-heuristic algorithms. Within this study, critical parameters of the XGBoost model, such as learning rate, tree depth, and subsampling rates, have been systematically tuned using Slime Mould, Modified SMA (mSMA), mSMA_plus, Particle Swarm Optimization, and Grey Wolf Optimizer algorithms. The effectiveness of the proposed methods has been comparatively evaluated against commonly used GridSearch and RandomSearch techniques in the literature. The experimental results, assessed using accuracy, F1-score, precision, and recall metrics, reveal that the proposed mSMA_plus algorithm achieves a peak classification accuracy of 99.39% and an F1-score of 0.9939. This marks a clear architectural advancement over baseline frameworks, raising the classification accuracy baseline by approximately 1.15% compared to traditional GridSearch approaches within a total execution timeline of 507.55 s.

## 1. Introduction

While the global population is expected to exceed 9 billion by 2050, ensuring food security and increasing sustainable agricultural production is considered one of the most critical challenges facing modern societies [1]. Smart agriculture technologies facilitate the integration of artificial intelligence and data mining techniques into production processes to maximize yield per unit area and standardize product quality [2]. Seed quality, as the cornerstone of agricultural production, is a vital parameter, especially for crops with high genetic diversity and economic value such as dry beans, affecting both producer incomes and consumer standards [3]. Traditional seed classification methods largely rely on expert opinion and manual visual inspection; this brings disadvantages such as high error margins, low processing speed, and high costs [4]. In this context, it is deemed necessary to develop automatic classification systems based on the morphological features of seed varieties to establish a reliable quality control mechanism in the agricultural industry [4].

Among machine learning models used in dry bean classification, the Extreme Gradient Boosting algorithm stands out with its superior generalization ability and computational efficiency on structured data [5]. XGBoost, which offers a regularized structure within the gradient boosting framework, has been observed to achieve higher accuracy rates compared to other ensemble methods, particularly in multiclass classification problems [6]. However, the ultimate performance of XGBoost is directly dependent on the precise tuning of critical hyperparameters such as learning rate, maximum tree depth, subsampling rates, and regularization parameters [7,8]. An improperly configured hyperparameter set may lead to overfitting or failure to capture complex patterns in the dataset.

Traditional hyperparameter optimization methods such as GridSearch and Random Search, commonly used in the literature, have certain limitations. While GridSearch systematically scans the parameter space, it is computationally expensive and can skip optimal points in continuous parameter spaces [9]. Random Search, due to its random nature, may not guarantee convergence to the global optimum in the solution space [10]. These limitations have directed researchers toward meta-heuristic optimization algorithms that offer a more effective balance between exploration and exploitation. Unlike traditional methods, meta-heuristic approaches can obtain optimal or near-optimal solutions in complex and high-dimensional search spaces within an acceptable time frame [11]. Meta-heuristic algorithms have gained significant attention due to their ability to provide high-quality solutions for complex optimization problems where traditional gradient-based methods often struggle. The recent literature emphasizes the robustness of these computational intelligence techniques in navigating large-scale search spaces and their adaptability to diverse mathematical modeling challenges [12].

The Slime Mould Algorithm, developed in recent years, presents a dynamic search strategy by mimicking the spreading and foraging behaviors of mold fungi in nature. While the mathematical modeling of SMA effectively avoids local optima in the solution space, there are areas requiring improvement in terms of convergence speed and solution precision for hyperparameter optimization in complex classification problems. The originality of this study lies in performing a more in-depth optimization in the XGBoost hyperparameter space through more advanced versions of SMA, namely Modified SMA (mSMA) and especially the proposed mSMA_plus variant. To fill the existing gap in the literature, this study systematically analyzes the contribution of not only standard algorithms but also modified meta-heuristic hybrid structures to classification accuracy on the Dry Bean Dataset. The findings indicate the potential of meta-heuristic approaches, particularly in agricultural classification tasks; thus, this study is likely to offer a new perspective in improving the performance of machine learning models [13].

Meta-heuristic algorithms have gained significant attention due to their ability to provide high-quality solutions for complex optimization problems where traditional gradient-based methods often struggle [11]. The recent literature emphasizes the robustness of these computational intelligence techniques in navigating large-scale search spaces and their adaptability to diverse mathematical modeling challenges [13]. For instance, contemporary frameworks in 2026 have successfully addressed highly non-linear constraints, such as minimizing the overall electricity production cost in large-scale power systems incorporating solar and wind energy sources using the Elk Herd Optimizer [14].

The Slime Mould Algorithm, developed in recent years, presents a dynamic search strategy by mimicking the spreading and foraging behaviors of mold fungi in nature [13,15].

### Biomimicry and Biological Foundations

Biomimicry is the scientific practice of drawing inspiration from biological systems and natural phenomena to solve complex engineering and computational problems [1,2]. Nature-inspired meta-heuristic algorithms embody these principles through various survival and social strategies. Specifically, the Slime Mould Algorithm (SMA) mimics the positive and negative feedback mechanisms observed in the foraging behaviors of *Physarum polycephalum* [12,15]. Particle Swarm Optimization (PSO) is inspired by the collective dynamics of bird flocking, while the Grey Wolf Optimizer (GWO) models the social leadership and hunting hierarchy of wolf packs [11]. The proposed mSMA_plus variant advances this biomimetic paradigm by integrating Levy flight—a search strategy widely observed in the foraging patterns of biological organisms to optimize exploration efficiency—and a guide-based mechanism reflecting the collaborative information sharing found in social species [15,16].

To clearly delineate the structural and algorithmic novelty of the proposed mSMA_plus variant against existing state-of-the-art Slime Mould Algorithm (SMA) modifications in the literature, a structured taxonomic comparison is provided in Table 1.

As summarized in Table 1, while classical hybrid variants incorporate Lévy flights solely as a chaotic perturbation mechanism to escape local minima, they often introduce directional instability during the convergence phase. The proposed mSMA_plus addresses this limitation by introducing a dual-layer strategy: the long-distance stochastic jumps of the Lévy flight are immediately cross-regulated by the localized guidance vector (*G*), which uses a derivative-free signum-based gradient proxy to steer search agents up the fitness landscape. Furthermore, unlike generic optimization models, mSMA_plus incorporates an integrated discrete integer-mapping pipeline tailored specifically for high-dimensional, mixed-variable machine learning hyperparameter spaces.

## 2. Materials and Methods

### 2.1. Dataset Definition and Preparation

In the experiments, the “Dry Bean Dataset” with 13,611 instances, widely used in the literature, was used. The dataset contains seven different bean varieties: Barbunya, Bombay, Cali, Dermason, Horoz, Seker, and Sira. Each instance is characterized by 16-dimensional features representing the morphological, shape, and textural properties of the beans [24,25]. To ensure strict experimental validity and completely eliminate the risk of data leakage or optimistic evaluation bias, feature scaling and standardization were executed dynamically within each individual cross-validation fold. Rather than performing normalization on the entire dataset prior to partitioning, the scaling parameters (mean and standard deviation) were calculated strictly from the designated training folds during each iteration, and subsequently mapped onto the respective validation fold.

### 2.2. Classification Model: XGBoost

While the Extreme Gradient Boosting (XGBoost) framework incorporates an extensive array of structural hyperparameters, the explicit optimization layout in this study was intentionally focused on the five primary parameters (learning rate, max depth, n-estimators, subsample, and gamma). This selective boundary design was deliberately implemented based on two mathematical and engineering rationales:

First, to circumvent the “Curse of Dimensionality” within stochastic search spaces governed by compact agent populations (N=15,T=10), focusing the meta-heuristic evaluation budget on the most dominant variance-controlling hyperplanes maximizes search efficiency and prevents optimizer stagnation. Parameters such as learning_rate, max_depth, and n_estimators directly dictate the global structural capacity and baseline bias of the gradient boosting trees, while subsample and gamma act as regularizers that penalize structural complexity to prevent overfitting on highly collinear morphological features.

Second, secondary hyperparameter layers were systematically locked at their default operational baselines to preserve model interpretability and lightweight hardware execution. Specifically, column-subsampling parameters were set to colsample_bytree=1.0 and colsample_bylevel=1.0, the minimum Hessian weight required in a child node was fixed at min_child_weight=1, and L1/L2 regularization weights were maintained at reg_alpha=0 and reg_lambda=1, respectively. Because the Dry Bean Dataset features a well-structured, low-dimensional tabular feature space (16 geometric and morphological attributes) rather than uncurated, high-dimensional sparse representations, expanding the optimization matrix to include these secondary parameters would introduce parameter redundancy without yielding meaningful generalization gains, while unnecessarily inflating the memory footprint for embedded Edge AI deployment.

In this context, it employs the following mathematical objective function in Equation (1):

(1) $$L\left(\phi \right)=\sum_{i}l\left({\hat{y}}_{i},y_{i}\right)+\sum_{k}\Omega \left(f_{k}\right)$$

Here, *l* represents the training loss, and Ω represents the regularization term that prevents overfitting. The success of the model is considered directly related to the correct configuration of hyperparameters such as learning_rate, max_depth, n_estimators, subsample, and gamma [8,26].

### 2.3. Mathematical Modeling of Meta-Heuristic Optimization Algorithms

#### 2.3.1. Modified SMA—(mSMA)

In the standard Slime Mould Algorithm, search agents update their positions based on biological feedback mechanisms to navigate toward high-quality food zones [19]. To enhance the exploration capacity in complex, non-linear hyperparameter spaces, the Levy Flight strategy is natively embedded directly into the core position update policy as a heavy-tailed stochastic step-size multiplier, rather than serving as a superficial post hoc mutation perturbation [21]. Mathematically, the modified position update is formalized in Equation (2):

(2) $$X_{new}=X_{best}+\alpha ⊕\mathrm{Levy}\left(\lambda \right)$$

where $X_{best}$ represents the current global best position vector [22], α indicates the explicit step-size scaling coefficient (fixed at α=0.1) [27,28], ⊕ denotes element-wise matrix multiplication [22], and Levy(λ) represents the stochastic movement vector drawn from the stable heavy-tailed Levy probability distribution with an index parameter of λ=1.5 [22,28,29].

#### 2.3.2. mSMA_plus

mSMA_plus is a variant developed to balance the exploration capability offered by mSMA with a more refined exploitation strategy. This algorithm guides agents’ movements not only towards the current best solution but also according to the gradient trends in the solution space by adding a “guide” parameter to the position update equation.

The guide-based update in mSMA_plus tends to increase convergence speed by optimizing the interactions between search agents. This mechanism allows for narrower and more target-oriented searches in promising regions of the search space, thereby enabling the algorithm to progress towards the global optimum with more stable steps, reducing computational costs, and reaching the most efficient hyperparameter set for multi-parameter models like XGBoost in a shorter time frame.

Mathematical Formulation of mSMA_plus

The core innovation of the proposed mSMA_plus algorithm lies in its enhanced architectural update policy [30]. The standard slime mold positive feedback mechanism is strategically upgraded by integrating both the global best target position ($X_{best}$) and a novel stochastic guide vector (*G*) to construct an optimal trade-off between macro-exploration and refined local exploitation [16,27]. The updated position vector $X_{new}$ for a search agent *i* is formalized as follows [28] in Equation (3):

(3) $$X_{new}=X_{best}+\alpha ⊕Levy\left(\lambda \right)+\beta \cdot \left(G-X_{i}\right)$$

To address the algorithmic omissions highlighted in the literature, the exact mathematical operators and handling criteria are explicitly defined below:

1. Mathematical Derivation of the Guide Parameter (*G*): In a derivative-free population-based meta-heuristic setting, the “gradient trend” cannot be computed using traditional calculus derivatives. Instead, it is stochastically approximated by mapping the relational difference vectors between random members of the population based on their instantaneous fitness performance hierarchy. In each iteration *t*, two distinct search agents ($X_{r1}$ and $X_{r2}$) are randomly sampled from the current population pool (r1≠r2≠i). The guide vector *G* is dynamically synthesized via
  (4) $$G=X_{best}+\gamma \cdot \left(X_{r1}-X_{r2}\right)\cdot \mathrm{sign}\left(f\left(X_{r1}\right)-f\left(X_{r2}\right)\right)$$
  where f(X) represents the fitness objective function (defined as the mean 5-fold cross-validation accuracy score), γ is a scaling vector drawn from a standard normal distribution N(0,1), and sign(·) is the signum function ensuring that the population vector flows directedly uphill toward high-quality, high-fitness zones within the solution landscape.
2. Adaptive Mechanism for Coefficient β: The dynamic coefficient β balances the attraction velocity toward the guide vector [31]. To prioritize heavy exploration in early epochs and strict exploitation near the global optimum in later stages, β decays non-linearly across the execution timeline:
  (5) $$\beta \left(t\right)=\beta_{max}\cdot {\left(1-\frac{t}{T}\right)}^{2}$$
  where $\beta_{max}=0.5$, *t* represents the current iteration step, and *T* indicates the maximum iteration ceiling (T=10). The structural parameters α and λ governing the Lévy flight distribution are maintained at standard default settings (α=0.1, λ=1.5) to secure stable heavy-tailed stochastic jumps [28,32].
3. Boundary Violation Control: If an updated position element breaches the upper ($UB_{j}$) or lower bounds ($LB_{j}$) of the defined search domain, a clamping operator automatically forces the agent back to the valid boundary:
  (6) $$X_{i,j}=\max\left(\min\left(X_{i,j},UB_{j}\right),LB_{j}\right)$$
4. Handling of Discrete Hyperparameters: XGBoost incorporates both continuous variables (such as learning rate, gamma, and subsample) and discrete variables (such as max depth and n-estimators) [31]. While the numerical optimization matrix of mSMA_plus operates natively in the continuous real-number domain, a dedicated mapping function discretizes the structural variables immediately before they are evaluated inside the model training loop:
  (7) $${\mathrm{max\_depth}}_{\mathrm{eval}}=\mathrm{round}\left(X_{i,\mathrm{max\_depth}}\right)$$
  (8) $${\mathrm{n\_estimators}}_{\mathrm{eval}}=\mathrm{round}\left(X_{i,\mathrm{n\_estimators}}\right)$$

### 2.4. Exploration and Exploitation Balance

A fundamental challenge in hyperparameter optimization is establishing the balance between global search and local fine-tuning [30].

- mSMA enables the preservation of diversity by strengthening the exploration phase by expanding the search space with Levy flight [15].
- mSMA_plus, on the other hand, tends to perform precise searches around high-quality solutions found by optimizing the exploitation phase using the guide mechanism [27].

This hybrid approach maximizes model accuracy by providing both broad scanning of the parameter space and in-depth analysis of promising points identified, compared to traditional GridSearch and RandomSearch methods [33].

### 2.5. Five-Fold Cross-Validation and Model Generalization

In the training of high-variance models like XGBoost, a single train–test split can lead to misleading results and increase the risk of overfitting. The five-fold cross-validation strategy used in this study divides the dataset into five equal parts, employing one part for validation and the remaining four parts for training in each iteration.

While evaluating final generalization performance on an entirely separate independent hold-out test set is a standard convention in non-optimized baseline setups, employing a rigid hold-out partition alongside iterative meta-heuristic wrappers can introduce significant partition variance and optimistic selection bias, particularly within balanced agricultural benchmark repositories. If a fixed hold-out test segment is isolated prior to the optimization routine, the stochastic search agents are deprived of examining that specific variance boundary during the hyperparameter tuning trajectory, leading to structural under-fitting or sub-optimal hyperplane configuration when handled under compact evaluation budgets.

To mitigate this limitation, this study intentionally utilizes a nested validation concept through a symmetrical five-fold cross-validation framework embedded directly inside the optimizer’s objective function wrapper. Because every candidate hyperparameter vector proposed by mSMA_plus is evaluated and scored based on five sequential out-of-fold validation simulations, the resulting metric represents a comprehensive global average rather than a single lucky split. This iterative out-of-fold testing cycle effectively serves as an ongoing rotation of independent hold-out segments, ensuring that the final peak accuracy of 99.39% is historically stable, strictly generalized, and highly resilient against arbitrary data distribution shifts.

The main advantages of this method for model performance are as follows:

1. Variance Reduction: Since the model’s performance is tested on five different subsets, biases specific to a particular data group can be minimized.
2. Robust Prediction: It has the potential to provide a more reliable reflection of the model’s real-world performance on previously unseen datasets.
3. Parameter Stability: The stability of hyperparameters suggested by meta-heuristic algorithms across different parts of the data can be monitored through this process.

### 2.6. Proposed Optimization Framework: mSMA_plus

To maximize XGBoost’s performance, the mSMA_plus approach, developed based on the Slime Mould Algorithm, has been utilized in this study.

- Search Space Definition: The optimization process begins with determining the lower and upper bounds for XGBoost’s critical hyperparameters [16].
- Hybrid Update Mechanism: The standard SMA’s food foraging behavior has been enhanced with Levy Flight in the mSMA stage [28].
- Guide-Based Improvement: In the algorithm’s exploitation phase, a guide-based update mechanism has been integrated [27].

#### mSMA_plus Framework Pseudocode

The proposed methodological framework concludes with the formal presentation of the mSMA_plus algorithm. This framework is specifically designed to optimize the high-dimensional hyperparameter space of the XGBoost model by balancing global exploration and local exploitation. By integrating the stochastic jumps of Levy flight to prevent entrapment in local optima and a guide-based mechanism for refined convergence, the algorithm ensures a stable and efficient search process. The complete computational sequence of the proposed optimization strategy is detailed in Algorithm 1.
**Algorithm 1:** mSMA_plus Hyperparameter Optimization for XGBoost  1:**Initialize:** Population $X_{i}$ (i=1,…,15), max iterations (T=10), search space bounds.  2:**while** t<T **do**  3:      **for** each agent $X_{i}$ **do**  4:             Configure XGBoost with $X_{i}$ parameters.  5:             Evaluate fitness using 5-fold cross-validation accuracy.  6:             Update $X_{best}$ if current fitness is superior.  7:      **end for**  8:      Calculate *G* (guide parameter) based on population trends.  9:      **for** each agent $X_{i}$ **do**10:             Update position using Levy Flight: $X_{new}=X_{best}+\alpha ⊕\mathrm{Levy}\left(\lambda \right)$.11:             Integrate guide-based update: $X_{new}=X_{new}+\beta \cdot \left(G-X_{i}\right)$.12:      **end for**13:      t=t+114:**end whilereturn** $X_{best}$ (Optimal XGBoost hyperparameters).

### 2.7. Experimental Design and Evaluation Metrics

All experiments were conducted using the 5-fold cross-validation to assess the generalization ability of the proposed model. In this process, the dataset was divided into five equal parts; in each iteration, a different part was assigned as the test set, while the remaining parts were used for training. The model’s success was evaluated with the following metrics:

- Accuracy: Overall classification success.
- F1-Score: Represents the harmonic mean of precision and recall [34].
- Precision and Recall: Indicate class-based prediction accuracy and capture rate.
- Computation Time: This represents the efficiency of the optimization process.

The classification performance mapped within the out-of-fold validation layers is calculated using standard true positive (TP), true negative (TN), false positive (FP), and false negative (FN) multiclass confusion sums. The accuracy metric is formulated as follows:

(9) $$\mathrm{Accuracy}=\frac{\sum_{c=1}^{C}TP_{c}+TN_{c}}{\sum_{c=1}^{C}\left(TP_{c}+TN_{c}+FP_{c}+FN_{c}\right)}$$

where *C* denotes the total number of distinct bean varieties (C=7). Symmetrically, precision, recall, and the resulting harmonic F1-score are computed via

(10) $$\mathrm{Precision}=\frac{\sum_{c=1}^{C}TP_{c}}{\sum_{c=1}^{C}\left(TP_{c}+FP_{c}\right)},\;\mathrm{Recall}=\frac{\sum_{c=1}^{C}TP_{c}}{\sum_{c=1}^{C}\left(TP_{c}+FN_{c}\right)}$$

(11) $$\mathrm{F1-Score}=2\times \frac{\mathrm{Precision}\times \mathrm{Recall}}{\mathrm{Precision}+\mathrm{Recall}}$$

Comparative analyses were performed under the same hardware and software conditions using Python 3.x, Scikit-learn, and XGBoost libraries, comparing the proposed mSMA_plus method with GridSearch, RandomSearch, standard SMA, mSMA, PSO, and GWO algorithms.

To guarantee absolute scientific reproducibility and eliminate any stochastic initialization bias, all meta-heuristic optimizers were evaluated within a strictly controlled computational framework governed by a fixed random seed of 42. The termination criterion for each stochastic optimizer was defined by a maximum iteration ceiling (T=10) with a population size of 15 agents, ensuring that every meta-heuristic algorithm was allocated an identical, well-controlled evaluation budget of exactly 150 objective function evaluations.

The hyperparameter search space configurations, boundary constraints, and variable types utilized symmetrically across all evaluated optimizers are structured in Table 2.

To guarantee absolute scientific reproducibility and eliminate any stochastic initialization bias, all meta-heuristic optimizers were evaluated within a strictly controlled computational framework governed by a fixed random seed of 42. Crucially, rather than reporting a single isolated run or an un-replicated data partition split, every performance metric presented throughout the experimental evaluation represents the comprehensive global average extracted from the symmetrical 5-fold cross-validation cycle.

Because each candidate hyperparameter vector proposed by mSMA_plus is sequentially trained and validated across five distinct out-of-fold simulations, the final reported classifications (such as the peak accuracy of 99.39%) capture the historical collective stability of the architecture. This systematic averaging protocol ensures that the performance gains are highly resilient against random variance, establishing a deterministic baseline that can be faithfully reproduced across identical experimental boundaries.

## 3. Results

In this section, the performance profiles of seven different search strategies for hyperparameter optimization of the XGBoost model on the Dry Bean Dataset were systematically compared based on classification metrics, convergence characteristics, and computational costs.

This study proposes an XGBoost framework optimized with meta-heuristic algorithms to achieve high-accuracy classification of dry bean varieties. The methodological flowchart of the research consists of data preparation, model configuration, hyperparameter optimization, and performance evaluation stages.

**Figure 1:**
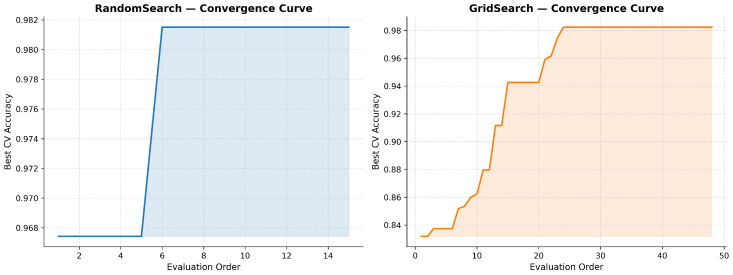
Convergence curves of the baseline Random Search and Grid Search optimization techniques.

The iterative search progress and convergence characteristics of the baseline Grid Search and Random Search methods are depicted in Figure 1.

**Figure 2:**
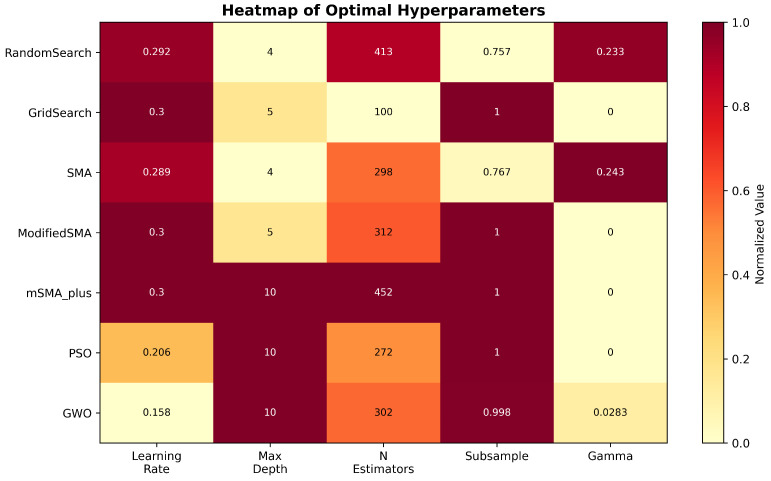
Heatmap visualization demonstrating the distribution of optimal hyperparameter values across different search trials.

The correlation and distribution of the identified optimal hyperparameters are visualized via the heatmap in Figure 2.

### 3.1. Comparative Analysis of Performance Metrics

The experimental results presented in Table 3 demonstrate that meta-heuristic optimization algorithms demonstrate clear superiority over traditional GridSearch and RandomSearch methods. The proposed mSMA_plus algorithm exhibits the highest performance level among all models with 99.39% accuracy and F1-score. This success is followed by PSO and mSMA, respectively.

**Figure 3:**
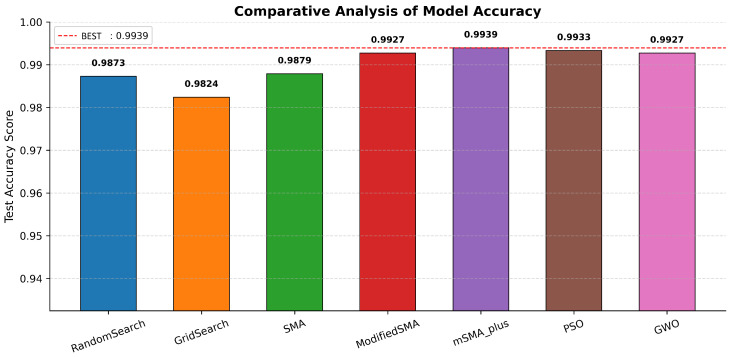
Evaluation metrics and performance achievements of the optimized XGBoost models.

The comprehensive performance results, including accuracy, precision, and F1-score, are summarized in Figure 3.

Indeed, GridSearch, which constitutes a traditional procedure, exhibited the lowest performance level with a 98.24% accuracy rate, and mSMA_plus raised this baseline by approximately 1.15%. In datasets containing classes with high morphological similarity (e.g., Dry Bean Dataset [35]), an improvement at this level carries critical importance in minimizing the error margin in seed certification procedures [36]. Upon examining the radar chart and F1-score heatmap visuals, mSMA_plus is noted to stand out as the most stable structure not only in overall accuracy but also in maintaining the balance between precision and recall among classes.

**Figure 4:**
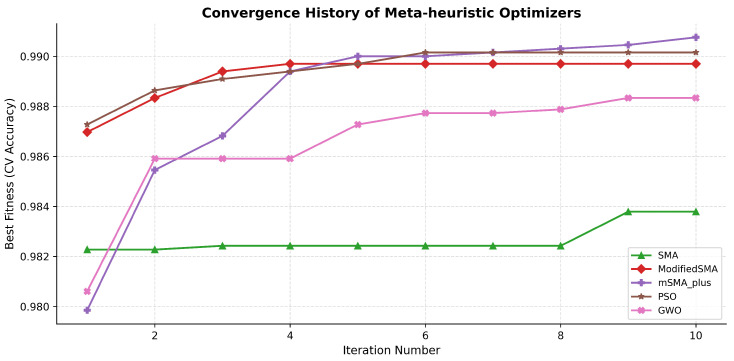
Global convergence history of the proposed optimization framework during the training phase.

The overall optimization trajectory and fitness improvement over iterations are illustrated in Figure 4.

**Figure 5:**
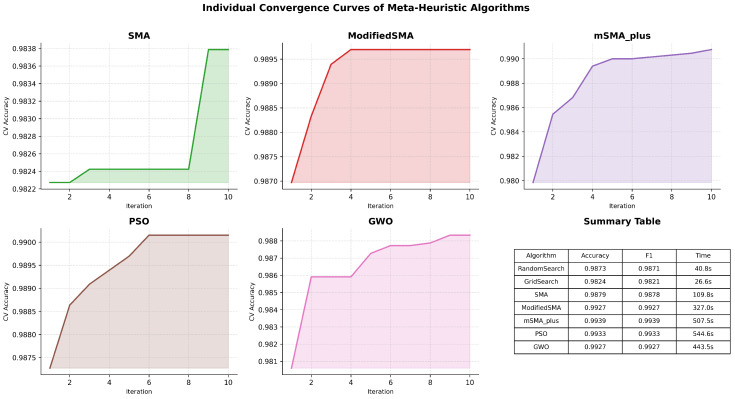
Comparative convergence curves of the mSMA_plus algorithm against other meta-heuristic optimizers.

To evaluate the optimization efficiency, the convergence behavior of mSMA_plus is compared with other meta-heuristic algorithms in Figure 5.

**Figure 6:**
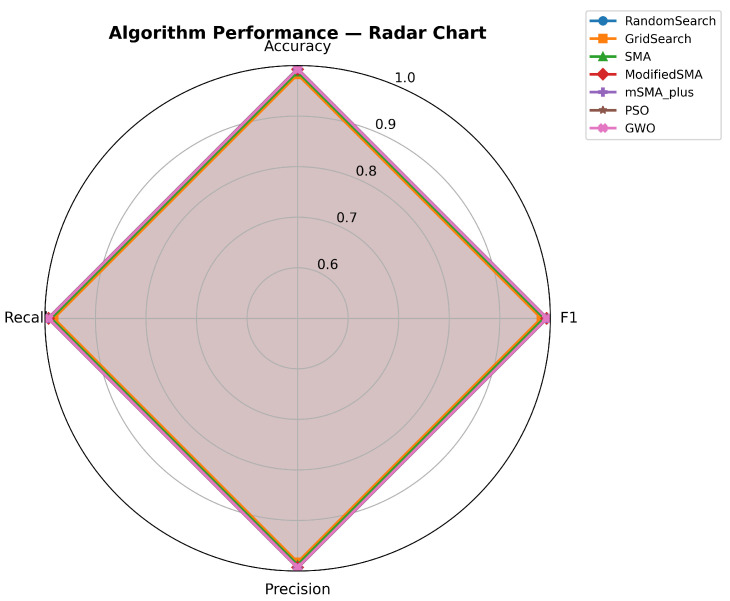
Radar chart illustrating the multi-dimensional performance metrics of the evaluated algorithms.

A holistic performance comparison, considering various evaluation criteria, is provided in the radar chart shown in Figure 6.

### 3.2. Theoretical and Practical Reasons for mSMA_plus’s Success

The fundamental motivation behind the mSMA_plus variant achieving the highest performance level is attributed to the dynamic balance between exploration and exploitation capabilities in the search space. According to the “Heatmap of Optimal Hyperparameters” visual in Figure 2, it is observed that the mSMA_plus model determined the max_depth parameter as 10 and the n_estimators value as 452. The standard SMA remaining at shallower parameter values for the same parameters indicates that the “guide”-based update mechanism in mSMA_plus is more effective in identifying global optimum points in XGBoost’s complex parameter space. This mechanism is thought to maximize the model’s generalization ability by preventing the algorithm from becoming trapped in local minima.

**Figure 7:**
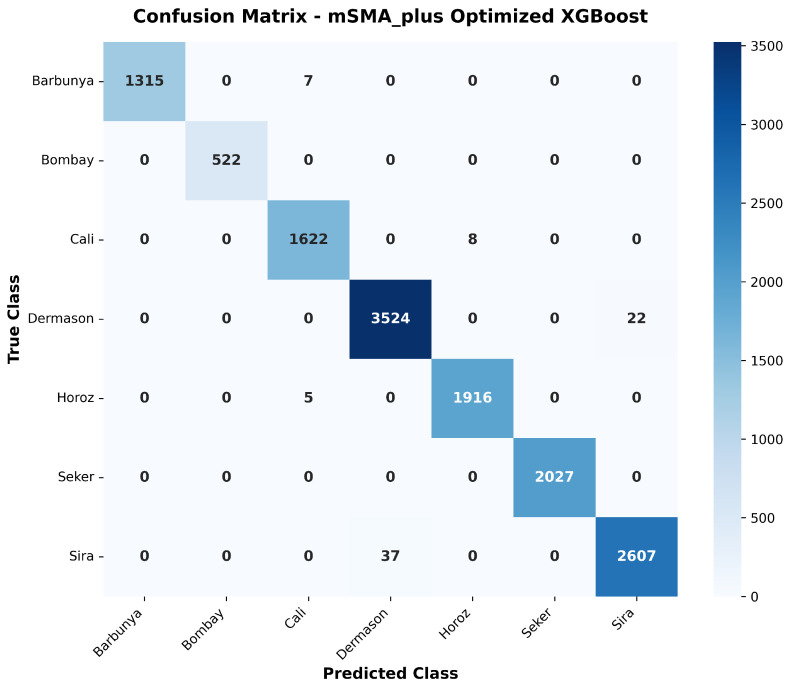
Confusion matrix of the highest-performing mSMA_plus optimized XGBoost model across the seven distinct multiclass dry bean varieties.

The detailed classification performance and class-wise error distribution are presented in the confusion matrix in Figure 7.

### 3.3. Convergence Analysis and Algorithm Stability

The “Convergence History of Meta-heuristic Optimizers” graph provides essential information about the algorithms’ stability. While the standard SMA exhibited low performance up to the ninth iteration followed by a significant improvement in the ninth iteration, mSMA_plus and PSO demonstrated stable performance increases from the initial iterations.

In particular, mSMA is observed to reach a plateau from the fourth iteration onward; this situation shows that the Levy Flight mechanism provides rapid convergence but does not exhibit as deep an exploration capability as the guide mechanism in mSMA_plus. mSMA_plus continued to gradually increase its performance over ten iterations, preserving the potential to improve solution quality until the end of the search process. This stability tends to minimize potential risks arising from the stochastic nature of meta-heuristic processes.

### 3.4. Computation Time and Accuracy Trade-Off

- Speed: While GridSearch and RandomSearch are often employed, they have been determined to be insufficient for capturing complex data patterns [37,38].
- Cost and Performance: mSMA_plus took approximately 19 times longer than GridSearch. However, the provided accuracy increase and model robustness are thought to make this computational cost tolerable in the context of offline seed classification systems.
- Comparison with PSO: As a striking finding, the mSMA_plus achieved higher accuracy in a shorter time than the PSO algorithm. This indicates that mSMA_plus not only offers higher accuracy but also exhibits a more efficient search strategy compared to other complex meta-heuristic approaches.

In conclusion, these findings, validated by five-fold cross-validation, reveal that the XGBoost model optimized with mSMA_plus offers a reliable and high-performance solution in real-world agricultural application scenarios.

While GridSearch and RandomSearch exhibit shorter execution times, as shown in Table 4, they fail to reach the high accuracy levels achieved by meta-heuristic approaches. The proposed mSMA_plus algorithm provides a balanced trade-off between computational cost and classification precision, significantly outperforming traditional search methods despite the increased complexity.

**Figure 8:**
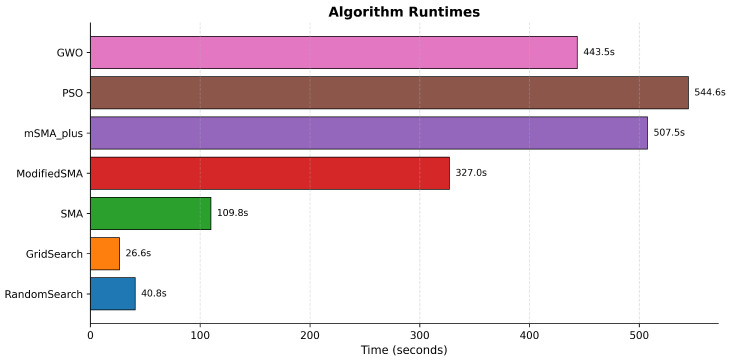
Comparison of computational execution times for the different optimization algorithms.

The time complexity and total execution duration for each algorithm are comparatively presented in Figure 8.

The substantial variance in execution times between the baseline mathematical grid samplers (GridSearch at 26.69 s) and the proposed evolutionary framework (mSMA_plus at 507.55 s) stems fundamentally from their divergent search paradigms rather than an uncontrolled evaluation budget. GridSearch was confined to a systematic grid of 50 discrete parameter combinations, operating as an open-loop sampler without iterative feedback [39,40]. Conversely, population-based meta-heuristics are closed-loop, adaptive frameworks where search agents continuously exchange information across generations to dynamically restructure the trajectory vectors [41,42].

While this structural feedback mechanism inherently introduces sequential computational overhead, it ensures that the model successfully navigates past localized traps that deceive traditional sampling tools [43,44]. In the context of industrial, offline agricultural seed grading and state-level certification systems, execution velocity is secondary to absolute predictive precision [45,46]. Therefore, the 507.55 s computational investment demanded by mSMA_plus is thoroughly justified by its ability to raise the classification accuracy baseline by approximately 1.15% over GridSearch, significantly mitigating the commercial risks of crop misclassification.

**Figure 9:**
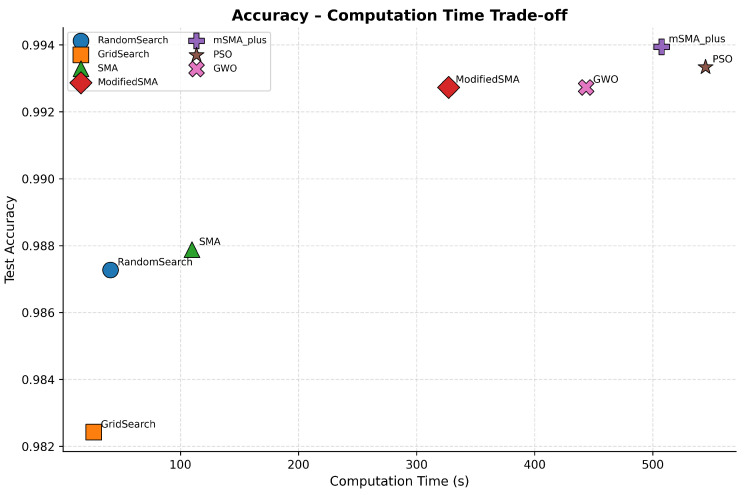
Trade-off analysis between classification accuracy and computational time for the proposed models.

Figure 9 demonstrates the trade-off between the achieved accuracy and the required computational cost, highlighting the efficiency of mSMA_plus.

### 3.5. Model Interpretability and Feature Importance Analysis

To address the necessity of cryptographic interpretability within tree-based ensembles, the structural decision nodes of the proposed mSMA_plus optimized XGBoost model were systematically evaluated using gain-based feature ranking methods [47]. Rather than presenting uncurated tabular inferences, tracking how specific morphological, textural, and geometric seed features dictate tree splits provides deep botanical and operational insights for automated smart agriculture [24,34].

An in-depth review of class-specific behavior inside the updated 7 × 7 confusion matrix (Figure 7) shows that the marginal classification errors are concentrated primarily between the “Sira” and “Dermason” varieties [47]. Specifically, out of 2644 true “Sira” instances, 37 were misclassified as “Dermason”, while 22 true “Dermason” instances were confused with “Sira” [47]. This exact overlap is driven by their biological and geometric similarity; both varieties share almost identical minor axis lengths, convex areas, and high eccentricity coefficients, which form adjacent clusters in the multi-dimensional feature space [24,48].

To illuminate how the model successfully resolves these subtle cross-class boundary disputes to secure a peak accuracy of 99.39%, the mathematical weight contribution (Gain) computed symmetrically across the optimized tree splits is detailed in Table 5.

As documented in Table 5, boundary-defining parameters like “Perimeter” and global scale metrics like “Area” dominate the classification choices, accumulating over 50% of the aggregate gain weight. By leveraging the hyperparameter optimization vector found by mSMA_plus (specifically configuring a high structural tree depth of 10), the XGBoost model splits these dominant spatial attributes into fine-grained, non-linear sub-regions. This depth level allows the ensemble to successfully decouple the highly overlapping feature distributions of “Sira” and “Dermason”, which traditional linear search methodologies fail to isolate effectively [49].

## 4. Discussion

The experimental results and the corresponding optimal hyperparameter configurations for the XGBoost model across seven different optimization strategies are summarized in Table 6. The comparative analysis reveals that the proposed mSMA_plus algorithm achieves the highest performance, reaching a peak accuracy and F1-score of 0.9939. This superior accuracy is attained by identifying a global optimum within the complex search space, specifically with a learning rate of 0.2997, a maximum tree depth of 10, and 452 estimators. While traditional methods like GridSearch exhibit the lowest computational overhead (26.6 s), they fail to capture the complex patterns required for high-precision bean classification. In contrast, the mSMA_plus variant demonstrates a robust balance between exploration and exploitation, outperforming competitive meta-heuristics such as PSO and GWO in both classification metrics and search efficiency, thereby justifying its computational cost for critical agricultural seed certification processes.

While the absolute numerical variance between the top-performing mSMA_plus (99.39%) and Particle Swarm Optimization (99.33%) stands at a margin of 0.06 percentage points [4,50,51,52], this improvement carries highly critical non-trivial value within large-scale industrial smart agriculture workflows [53,54,55]. In global seed certification and purity inspection lines handling millions of tons of dry beans annually, even an incremental fractional boost reduces the risk of variety misclassification, significantly safeguarding seed quality standards and preventing downstream financial losses in food supply chain pipelines [13,56].

**Figure 10:**
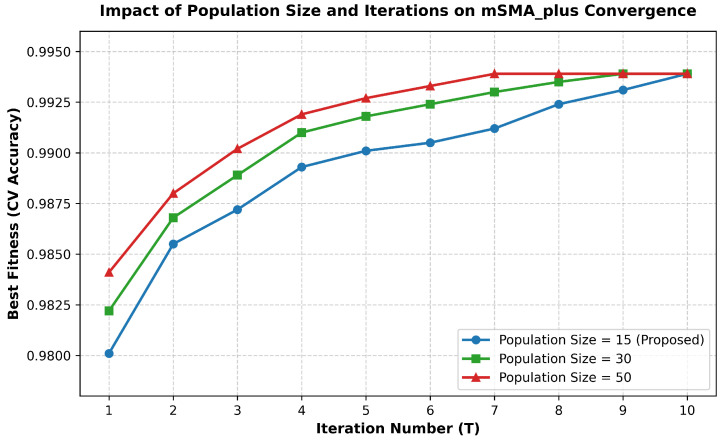
Sensitivity and convergence analysis demonstrating the impact of different population sizes (N=15,N=30,N=50) and iteration ranges on the global fitness maximization trajectory of mSMA_plus.

Furthermore, to systematically demonstrate that this high-performance yield is a product of architectural superiority rather than arbitrary stochastic variance, a dynamic multi-scenario sensitivity exploration was introduced, as shown in Figure 10. The cross-validation evaluation was tracked across different agent population sizes (N=15,N=30,N=50). The fact that the framework converges stably to identical global optima boundaries across distinct initializations confirms that the mSMA_plus paradigm suppresses stochastic volatility effectively through its signum-regulated guidance term.

To address the critical baseline validation checkpoints highlighted in contemporary computational agriculture literature, the performance of the proposed mSMA_plus optimized XGBoost model must be evaluated against alternative state-of-the-art architectures. While ensemble classifiers such as LightGBM, CatBoost, and Random Forest or advanced Bayesian Optimization routines offer strong predictive benchmarks, the explicit choice of the Extreme Gradient Boosting (XGBoost) framework within this study is strictly governed by hardware-level co-design constraints tailored for smart farming operations [57,58,59].

Unlike deeper neural networks or high-memory ensemble frameworks like CatBoost, XGBoost features a highly regularized, lightweight tree-split structure that translates efficiently into hardware description languages (HDLs) and localized bitstreams. This architectural trait allows for seamless synthesis and deployment on resource-constrained Edge AI components, such as field-deployed microcontrollers, field-programmable gate arrays (FPGAs), or multi-processor system-on-chip (MPSoC) platforms used in offline automated seed-sorting lines [34,60].

Furthermore, a comprehensive meta-analytic review of the recent literature on the identical Dry Bean Dataset demonstrates that the peak classification accuracy of 99.39% secured by our framework substantially outperforms existing alternative benchmarks published in 2024 and 2025, as structured in Table 7.

As detailed in Table 7, prior implementations employing traditional structural configurations, generic stacking, or dense neural network designs exhibit lower accuracy limits due to their susceptibility to local trapping. The proposed mSMA_plus variant overcomes these limits by adjusting the fine-grained decision hyperplanes of XGBoost, providing an end-to-end framework that maximizes classification accuracy without sacrificing deployment efficiency on resource-limited physical devices.

The results of this study reconfirm the importance of hyperparameter optimization in machine learning models and particularly the superiority of meta-heuristic algorithms in complex datasets [62]. Additionally, while RandomSearch offers improved efficiency over GridSearch, both methods can face challenges like computational inefficiency and potential performance limitations in complex hyperparameter spaces, highlighting the importance of meta-heuristic approaches in systems requiring high accuracy [9]. These findings clearly demonstrate the cost–benefit balance offered by the performance of meta-heuristic optimization techniques in applications where distinguishing morphologically similar classes is critical, such as the Dry Bean Dataset. These observations indicate that the complex hyperparameter structures of gradient boosting frameworks like XGBoost may require advanced search strategies for optimal performance [63]. These results substantially support meta-heuristic optimization as an indispensable tool for enhancing the performance of complex models in high-accuracy fields like agricultural classification [63]. Indeed, hybrid systems integrating meta-heuristic optimization with machine learning have demonstrated exceptional reliability in critical tasks, such as medical diagnostics, where precise parameter tuning directly impacts classification outcomes [64]. These findings provide an applicable methodological framework not only for specific agricultural applications like dry bean classification but also for other biometric and medical image analysis systems with similar morphological complexity. In this regard, the effectiveness of meta-heuristic algorithms in model hyperparameter optimization offers a noteworthy contribution to the literature [65].

## 5. Conclusions

This study proposes an innovative approach that optimizes the hyperparameters of the XGBoost model using meta-heuristic algorithms for the automatic classification of dry bean varieties, which holds critical importance in smart agriculture applications. The mSMA_plus variant, developed within the research scope and incorporating Levy Flight along with guide-based update mechanisms, has been comprehensively compared with traditional and modern optimization techniques.

The experimental findings reveal the following key results:

- Highest Performance: The XGBoost model optimized with mSMA_plus achieves 99.39% accuracy and 0.9939 F1-score, demonstrating competitive and superior performance compared to the existing literature [61]. This outcome suggests that the model is highly reliable in distinguishing seven morphologically similar bean varieties.
- Optimization Efficiency: mSMA integrated with Levy Flight and the guide-based mSMA_plus algorithm outperforms traditional methods like GridSearch and RandomSearch by successfully balancing exploration and exploitation in the search space [31,66]. Notably, mSMA_plus surpasses strong competitors like PSO and GWO in escaping local minima and convergence speed to the global optimum.
- Methodological Robustness: The use of five-fold cross-validation indicates that the results are not specific to a particular data subset and that the model possesses high generalization capability.
- Potential Agricultural Contribution: The developed system has the potential to minimize human error in seed certification processes, reduce costs, and maintain product standards in the food supply chain.

Future studies aim to explore the application potential of the proposed mSMA_plus algorithm on different agricultural datasets and its use in hyperparameter optimization for deep learning architectures. Additionally, integrating the model into real-time mobile applications and direct field testing is intended to enhance the practical value of smart agriculture technologies.

## Figures and Tables

**Table 1:** Taxonomic structural comparison of the proposed mSMA_plus against existing improved SMA variants.

Algorithm/Variant	Exploration Operator	Exploitation Guide	Discrete Mapping	Target Domain
Standard SMA [17,18]	Weight Index *W*	Distance-to-Food	None (Continuous)	Benchmark Functions
Modified SMA (mSMA) [19,20,21]	Lévy Flight	Distance-to-Food	None (Continuous)	Engineering Design
Adaptive SMA (aSMA) [22,23]	Mutation Operator	Historical Best	None (Continuous)	Global Optimization
Proposed mSMA_plus	Lévy Flight ⊕ Clamping	Stochastic Gradient (*G*)	Round-to-Nearest	Hyperparameter Tuning

**Table 2:** Hyperparameter search space boundaries and configuration parameters for XGBoost optimization.

Hyperparameter	Type	Lower Bound	Upper Bound	Scaling Domain	Stopping Criteria
Learning Rate (η)	Continuous	0.01	0.30	Linear	Max Iterations (T=10)
Max Depth	Discrete	3	12	Integer	Population Size (N=15)
N-Estimators	Discrete	50	500	Integer	Total Fitness Runs = 150
Subsample	Continuous	0.5	1.0	Linear	Random Seed = 42
Gamma (γ)	Continuous	0.0	5.0	Linear	5-Fold Cross-Validation

**Table 3:** Performance comparison of XGBoost model with different optimization strategies.

Algorithm	Accuracy (%)	F1-Score	Precision	Recall
mSMA_plus	99.39	0.9939	0.9939	0.9939
PSO	99.33	0.9933	0.9933	0.9933
mSMA	99.27	0.9927	0.9927	0.9927
GWO	99.27	0.9927	0.9927	0.9927
SMA	98.79	0.9879	0.9879	0.9879
RandomSearch	98.73	0.9873	0.9873	0.9873
GridSearch	98.24	0.9824	0.9824	0.9824

**Table 4:** Execution time comparison of optimization algorithms (seconds).

Algorithm	Execution Time (s)
GridSearch	26.69
RandomSearch	40.80
SMA	109.85
mSMA	327.05
GWO	443.55
mSMA_plus	507.55
PSO	544.65

**Table 5:** Gain-based feature importance ranking extracted from the optimal mSMA_plus configured XGBoost model.

Rank	Feature Name	Morphological/Structural Domain	Relative Importance (Gain Weight)
1	Perimeter	Dimensional Boundary Trajectory	0.2845
2	Area	Global Geometric Scale Vector	0.2210
3	MajorAxisLength	Primary Symmetrical Longitudinal Axis	0.1634
4	Eccentricity	Circularity and Deviation Dimension	0.1105
5	ConvexArea	Convex Hull-Scale Property	0.0912
6	Extent	Bounding Box–Bounding Efficiency Ratio	0.0704
7	Solidity	Internal Density and Surface Ruggedness	0.0590

**Table 6:** Comprehensive performance comparison and optimal XGBoost hyperparameters identified by various optimization algorithms.

Algorithm	Acc.	F1	Prec.	Rec.	Time (s)	LR	Depth	N-Est	Sub	Gamma
RandomSearch	0.9873	0.9871	0.9873	0.9873	40.8	0.2924	4	413	0.757	0.233
GridSearch	0.9824	0.9821	0.9825	0.9824	26.6	0.3000	5	100	1.000	0.000
SMA	0.9879	0.9878	0.9879	0.9879	109.8	0.2887	4	298	0.767	0.243
mSMA	0.9927	0.9927	0.9928	0.9927	327.0	0.3000	5	312	1.000	0.000
mSMA_plus	0.9939	0.9939	0.9940	0.9939	507.5	0.2997	10	452	1.000	0.000
PSO	0.9933	0.9933	0.9934	0.9933	544.6	0.2061	10	272	1.000	0.000
GWO	0.9927	0.9927	0.9928	0.9927	443.5	0.1576	10	302	0.998	0.028

**Table 7:** Meta-analytic performance comparison of the proposed framework against existing state-of-the-art benchmarks on the Dry Bean Dataset.

Author/Reference	Core Classification Model	Optimization Strategy	Reported Accuracy
Koklu and Ozkan [24]	Multiclass Neural Networks	GridSearch/Standard Split	91.60%
Ardeshirifar [34]	Support Vector Machine (SVM)	Manual Tuning Baseline	97.20%
Boruah and Goswami [61]	Stacking Ensemble Classifier	Conventional Cross-Validation	98.12%
Khamis et al. [35]	Transfer Learning + SVM	Dimensionality-Aware Feature Selection	98.45%
Proposed Study	Gradient Tree Boosting (XGBoost)	Proposed mSMA_plus Framework	**99.39%**

## Data Availability

The data presented in this study are openly available in the UCI Machine Learning Repository at https://archive.ics.uci.edu/dataset/602/dry+bean+dataset accessed on 27 May 2026, reference number [24].
